# Generation of Triple-Transgenic *Forsythia* Cell Cultures as a Platform for the Efficient, Stable, and Sustainable Production of Lignans

**DOI:** 10.1371/journal.pone.0144519

**Published:** 2015-12-07

**Authors:** Jun Murata, Erika Matsumoto, Kinuyo Morimoto, Tomotsugu Koyama, Honoo Satake

**Affiliations:** Bioorganic Research Institute, Suntory Foundation for Life Sciences, Kyoto 619–0284, Japan; University of Illinois at Urbana-Champaign, UNITED STATES

## Abstract

Sesamin is a furofuran lignan biosynthesized from the precursor lignan pinoresinol specifically in sesame seeds. This lignan is shown to exhibit anti-hypertensive activity, protect the liver from damages by ethanol and lipid oxidation, and reduce lung tumor growth. Despite rapidly elevating demand, plant sources of lignans are frequently limited because of the high cost of locating and collecting plants. Indeed, the acquisition of sesamin exclusively depends on the conventional extraction of particular *Sesamum* seeds. In this study, we have created the efficient, stable and sustainable sesamin production system using triple-transgenic *Forsythia koreana* cell suspension cultures, U18i-CPi-Fk. These transgenic cell cultures were generated by stably introducing an RNAi sequence against the pinoresinol-glucosylating enzyme, UGT71A18, into existing CPi-Fk cells, which had been created by introducing *Sesamum indicum* sesamin synthase (CYP81Q1) and an RNA interference (RNAi) sequence against pinoresinol/lariciresinol reductase (PLR) into *F*. *koreanna* cells. Compared to its transgenic prototype, U18i-CPi-Fk displayed 5-fold higher production of pinoresinol aglycone and 1.4-fold higher production of sesamin, respectively, while the wildtype cannot produce sesamin due to a lack of any intrinsic sesamin synthase. Moreover, red LED irradiation of U18i-CPi-Fk specifically resulted in 3.0-fold greater production in both pinoresinol aglycone and sesamin than production of these lignans under the dark condition, whereas pinoresinol production was decreased in the wildtype under red LED. Moreover, we developed a procedure for sodium alginate-based long-term storage of U18i-CPi-Fk in liquid nitrogen. Production of sesamin in U18i-CPi-Fk re-thawed after six-month cryopreservation was equivalent to that of non-cryopreserved U18i-CPi-Fk. These data warrant on-demand production of sesamin anytime and anywhere. Collectively, the present study provides evidence that U18i-CP-Fk is an unprecedented platform for efficient, stable, and sustainable production of sesamin, and shows that a transgenic and specific light-regulated *Forsythia* cell-based metabolic engineering is a promising strategy for the acquisition of rare and beneficial lignans.

## Introduction

**Fig 1:**
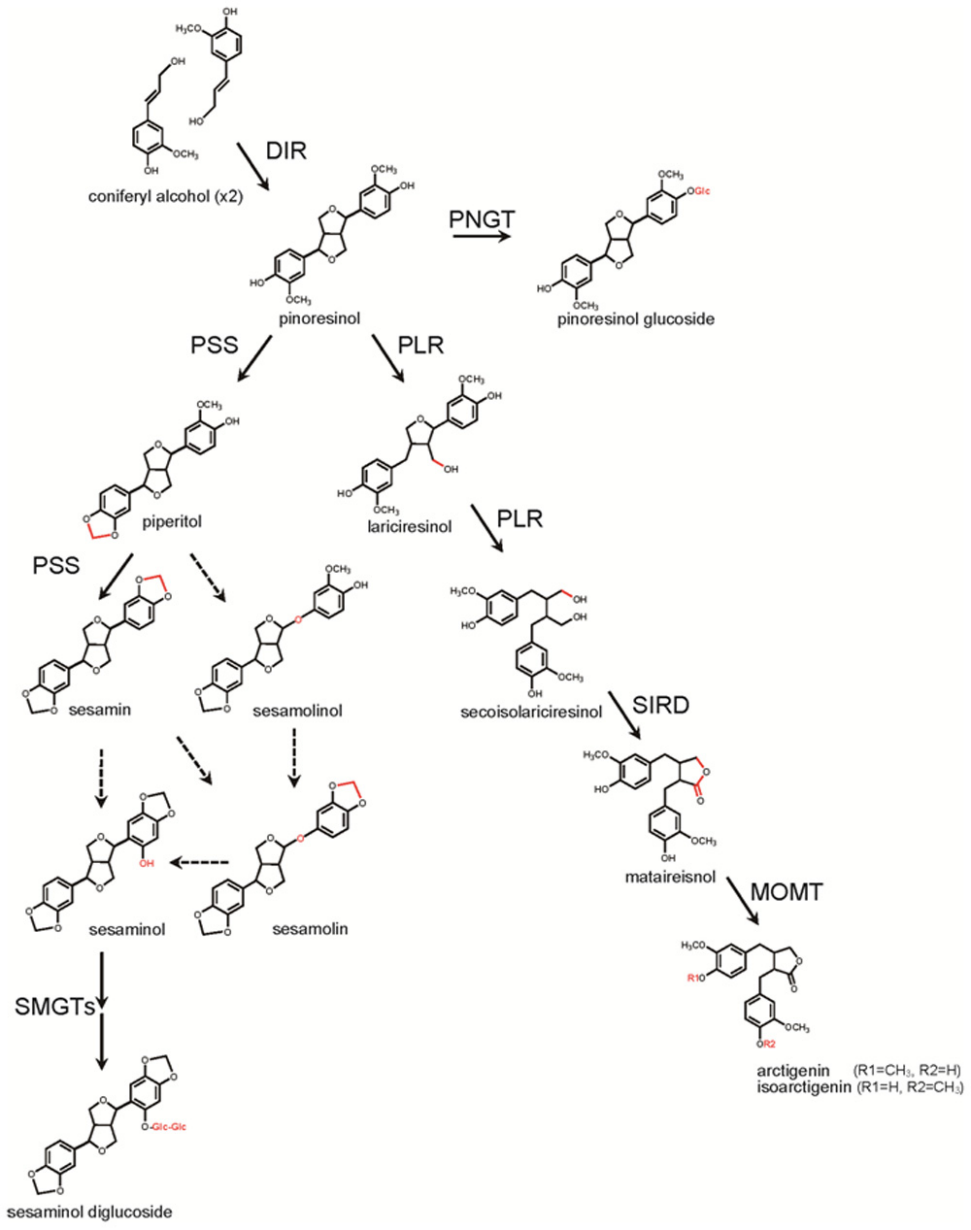
Biosynthesis pathways of major lignans in *Forsythia and Sesamum*. Chemical conversions at each step are indicated in red. Solid and broken lines represent identified and unidentified enzyme-catalyzed reactions, respectively.

The consistent and appropriate intake of low-cost healthy diets and clinical drugs are the most promising and effective ways to improve the quality of life including a healthy life expectancy and to prevent lifestyle-related diseases. Particularly, the recent escalation in the number of elderly individuals has increased the importance of efficient dietary supplement and drug development. Over the past few decades, plant specialized metabolites (formerly termed secondary metabolites), including alkaloids, flavonoids, isoflavonoids, and lignans, have attracted attention as dietary supplements and medicines. Lignans are naturally occurring phenylpropanoid dimers (C6-C3 units; e.g., coniferyl alcohol), in which the phenylpropane units are linked by the central carbons of the side chains [1–7]. Lignans have been characterized from *Podophyllum*, *Linum*, *Sesamum*, *Forsythia* and various other plant families [1–7]. Sesamin is classified as a furofuran lignan and is the most abundant water-insoluble lignan in *Sesamum indicum* (sesame) seeds [1–7]. This *Sesamum* lignan is biosynthesized by the sesame cytochrome P450, CYP81Q1, through the formation of two methylenedioxy bridges in a precursor lignan, pinoresinol (Fig 1) [5–8]. Sesamin was also shown to exert diverse beneficial effects on mammals including human [1–7], including an anti-hypertensive effect [9], the reduction of breast tumor growth [10], and recovery of liver damage caused by ethanol and lipid oxidation [11, 12]. These findings indicate that the demand for sesamin will rapidly increase in the near future. However, sesamin is acquired via extraction from sesame seed oil, and although sesame plants produce more sesamin than any other plant, the oil contains a maximum of 0.4–0.6% (w/w) sesamin [1–7]. Furthermore, sesame seeds are cultivated only once per year, thereby limiting opportunities to obtain large amounts of this compound. These shortcomings, combined with elevating demands on sesamin, indicate that a novel strategy for systematic sesamin production is clearly required.

Genetic engineering of biosynthetic pathways in plants and *in vitro* cell or organ cultures either through stable or transient transformation has been attempted in the development of production systems for several plant specialized metabolites, such as alkaloids and flavonoids [13–23]. However, most of transgenic metabolic engineering studies involve merely overexpression of, or RNA interference against, an endogenous biosynthetic enzyme. Furthermore, there have been no reports of the up-regulation of authentic and exogenous beneficial lignan production via either transient or stable gene engineering, except for production of sesamin using *Forsythia* cells [20]. Wild-type *Forsythia koreana* plant lacks *CYP81Q1* and its functional orthologs, and therefore fails to produce sesamin [1–7]. Instead, this plant abundantly and consistently produces a direct precursor of sesamin, pinoresinol [1–7, 20, 24], which is then either reduced stepwisely into secoisolariciresinol via pinoresinol-lariciresinol reductase (PLR) or glucosylated by UGT71A18 [1–7, 20, 25] in *Forsythia* (Fig 1). Previously, we showed that sesamin was produced by CPi-Fk, a double-transgenic cell suspension culture line of *F*. *koreana* in which a *Sesamum* sesamin-synthase *CYP81Q1* is constitutively expressed and an endogenous *PLR* is suppressed by RNAi [20]. This was the first report on the exogenous lignan via metabolic engineering, suggesting the potential of CPi-Fk as a prototype of the efficient, stable and sustainable sesamin production system. Unfortunately, CPi-Fk cannot be stored by any procedures, which is a critical drawback of CPi-Fk as a stable and sustainable platform of lignan production. Furthermore, the ability of CPi-Fk to produce sesamin was expected to be markedly improved by introduction of other genes, optimization of culture conditions, and/or the establishment of the procedure for long-term storage. In this paper, we present the development of a triple-transgenic *F*. *koreana* cell line, U18i-CPi-Fk, which exhibits a marked increase in production of pinoresinol aglycone and sesamin over that of CPi-Fk and the establishment of an optimized procedure for the long-term cryopreservation and recovery of U18i-CPi-Fk.

## Materials and Methods

### Cell culture

All cell suspension culture lines were maintained in Gamborg’s B-5 liquid medium supplemented with 6% sucrose and 0.05 mg l^-1^ 2,4-D (Cell Culture Medium; CCM) at 22°C. All suspensions were agitated on a rotary shaker at 110 rpm in the dark and subcultured every 2 weeks with an inoculum of 5 ml of saturated suspension cells. For light treatments, cell cultures were grown in CCM for 2 weeks either under continuous irradiation by blue LED (450–550 nm, 470 nm peak), red LED (600–700 nm, 630 nm peak) (NKsystem, Japan) or white light (white fluorescent tubes) at 100 μmol m^-2^s^-1^ photosynthetic photon flux density (PPFD) after an initial 2-week cultivation in the dark.

### Binary vector construction

To construct a binary vector for the RNAi of the *UTG71A18* gene (Accession: AB524718), the corresponding open reading frame was amplified by PCR using a primer set, RNAi-cacc-UGT71A18-F (5’-CAC CCA GGA ATA GGT CAC TTG ATATCAA-3’) and RNAi-UGT71A18-R (5’-GAA TGG AGC CAA CTA TCC TT-3’). The resultant PCR product was inserted into the pANDA35HK RNAi vector [26] using LR Clonase (Life Technologies).

### Transformation of suspension cells

A single colony of *Agrobacterium tumefaciens* strain EHA105 harboring the UGT71A18-RNAi vector was used to inoculate LB (Luria-Bertani) liquid medium containing 50 mg ml^-1^ kanamycin and grown at 28°C in a gyratory shaker (180 rpm) until OD600 reached 0.5. The cultures of *A*. *tumefaciens* (100 μl) were added to 10 ml of 4-day-old cultures of *F*. *koreana* CPi-Fk cells [20] in CCM, followed by co-incubation at 27°C for 2 days in the dark. The suspension cells were then washed four times with 10 ml of fresh CCM, and were plated onto solid CCM medium containing 50 mg ml^-1^ hygromycin and 100 mg ml^-1^ cefotaxime. Hygromycin-resistant callus were selected and transferred onto a fresh solid medium containing 50 mg ml^-1^ hygromycin, and then resuspended in CCM liquid medium to obtain suspensions of UGT71A18-RNAi-transformed CPi-Fk, designated as U18i-CPi-Fk. The resultant U18i-CPi-Fk suspension line was maintained by agitating at 120 rpm in the same medium at 25°C in the dark.

### Reverse transcription (RT)-PCR

Total RNA was isolated from 7-day-old suspension cells by the use of RNeasy Plant Mini Kit (Qiagen, CA). First-strand cDNA synthesis was performed using 1 μg of the total RNA with reverse transcriptase SuperScript III (Invitrogen, CA). The endogenous *PLR* (Accession: AAC49608), *UGT71A18* (Accession: AB524718), *rRNA* (Accession: AJ236041) and introduced *CYP81Q1* (Accession: AB194714) were amplified with the following primer sets: PLR-F (5'-ATG GGA AAA AGC AAA GTT TTG ATC ATT GG-3') and PLR-R (5'-CAC GTA ACG CTT GAG GTA CTC TTC CAC-3') for PLR, UGT71A18-F (5'-TAG CAG ATC AAC CCA GTA AAT-3') and UGT71A18-R (5'-TAG CAG ATC AAC CCA GTA AAT-3') and for UGT71A18, rRNA-F (5'-GAA ACC TGC AAA GCA GA-3'), rRNA-R (5'-CTG ACC TGG GGT CGC TGT CGA-3') for rRNA, and CYP81Q1-F (5'-ATG GAA GCT GAA ATG CTA TAT TCA GCT-3') and CYP81Q1-R (5'- AAC GTT GGA AAC CTG ACG AAG AAC TTT TTC-3') for CYP81Q1, respectively. PCR reactions with ExTaq DNA polymerase (Takara Bio, Shiga, Japan) were run at 94°C for 1 min followed by 28 cycles of 94°C for 30 sec, 57°C for 30 sec and 72°C for 1 min, and a final extension at 72°C for 7 min (GeneAmp 9700, Applied Biosystems). PCR products were visualized using 1.5% agarose gel electrophoresis by ethidium bromide staining.

### Lignan quantification

Lignan quantification was performed as previously reported [20, 24]. In brief, the suspension cells were cultured for 14 days, and frozen in liquid (LN_2_) nitrogen and lyophilized followed by extraction by 80% methanol at 4°C and evaporation *in vacuo*. The residue was dissolved in water, and the remaining aqueous phase, containing the lignan glycoside, was digested at 40°C overnight with 6 units/ml almond *β*-glucosidase (Sigma) in 0.15 M sodium acetate buffer (pH 5.2). The resulting samples were adjusted with 50% acetonitrile and then centrifuged at 15,000 rpm for 5 min. The supernatant was filtered through a Millex-LH filter (0.45 μm 4 mm^-1^; Millipore) and then subjected to analysis by reverse-phase high performance liquid chromatography (HPLC) using a Develosil C30-UG-5 column (4.6 × 150 mm, Nomura Chemical, Aichi, Japan). Each sample was eluted with a linear gradient of 35–90% solvent B [90% acetonitrile containing 0.1% (v/v) trifluoroacetic acid] in solvent A [H_2_O containing 0.1% (v/v) trifluoroacetic acid] for 20 min at a flow rate of 0.6 ml min^-1^ and then was eluted with 90% solvent B for 7 min. Lignans were monitored by UV absorption at 280 nm, and identified by both retention time and mass spectrum comparison with standard compounds [20].

### Stock of cells in liquid nitrogen

Cryopreservation of *F*. *koreana* wildtype and transgenic cells was tested under various conditions using sodium alginate based on previous reports with various modifications [27, 28]. U18i-CPi-Fk was centrifuged at 100 x *g* for 5 min, and was then resuspended in Gamborg’s B-5 medium containing 2% (w/v) sodium alginate, with cell density of 5 x 10^6^ / ml. The cell suspension was dropwisely added to Gamborg’s B-5 medium containing 0.1 M calcium chloride, leading to encapsulation of cells in alginate beads. The resultant beads were washed by Gamborg’s B-5 medium for 10 min, and incubated for 1 h at 25°C in Gamborg’s B-5 medium containing 2 M glycerol, 0.4 M sucrose, and 1% (w/v) proline. Five beads were transferred to a 1-ml cryovial in 0.25 ml of the same medium, followed by 5-hour pre-freezing at -30°C and storage in liquid nitrogen.

### Re-culturing of cells from cryopreservation

Cryopreserved cells were rapidly thawed at 40°C, and the beads were stepwisely incubated in Gamborg’s B-5 medium containing 1.2 M sucrose at 25°C for 15 min, Gamborg’s B-5 medium containing 0.5 M sucrose at 25°C for 15 min, and standard Gamborg’s B-5 medium. The recovered cells were re-grown for 55 days as described above and provided for the lignan quantification.

### Statistical analysis

Results are expressed as means ± SEM for the indicated number of observations. Data were analyzed by one-way ANOVA with Dunnett’s error protection. Differences were accepted as significant for *p* < 0.05.

## Results

### Generation of U18i- CPi-Fk

Initially, we attempted to simultaneously introduce CYP81Q1, PLR-RNAi, and UGT71A18-RNAi cDNA sequences into *F*. *koreana* wildtype cells using an expression vector bearing these three sequences. However, this approach failed to obtain triple-transgenic cell cultures, because the expression of either *PLR* or *UGT71A18* was not suppressed by RNAi in the resultant transgenic cells. We therefore introduced the UGT71A18-RNAi sequence into transgenic *F*. *koreana* cells harboring CYP81Q1 and PLR-RNAi (CPi-Fk) [20]. CPi-Fk was transformed with *A*. *tumefaciens* containing the UGT71A18-RNAi sequence. More than twenty cell lines were initially selected and grown on Gamborg’s B-5 solid media containing hygromycin and cefotaxime. Several independent cell lines, designated as U18i-CPi-Fk, were selected and proliferated in Gamborg’s B-5 liquid medium as suspension cultures.

**Fig 2:**
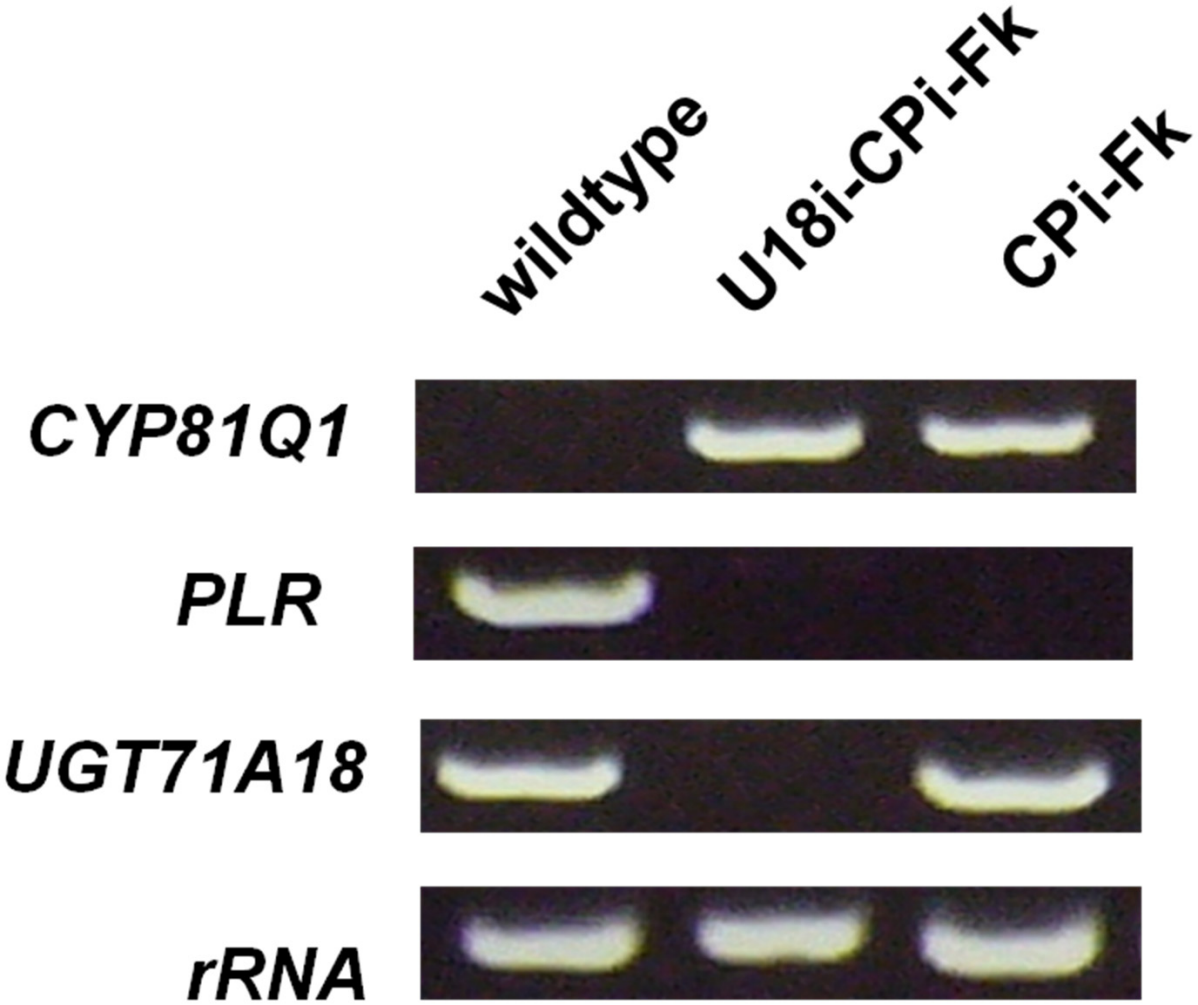
Generation of U18i-CPi-Fk. Expression of CYP81Q, PLR, and UGT71A18 in *F*. *koreana* wildtype, CPi-Fk, and U8i-CPi-Fk.

RT-PCR revealed that the expression of both *UGT71A18* and *PLR* was completely suppressed in a U18i-CPi-Fk line, compared to those of *F*. *koreana* wildtype cells (Fig 2). *CYP81Q1* gene expression was also observed specifically in U18i-CPi-Fk, as seen in CPi-Fk (Fig 2). Altogether, these results provide evidence that all three transgenes, *CYP81Q1*, PLR-RNAi, and UGT71A18-RNAi, were functionally expressed in U18i-CPi-Fk.

### Lignan production in U18i-CPi-Fk

**Fig 3:**
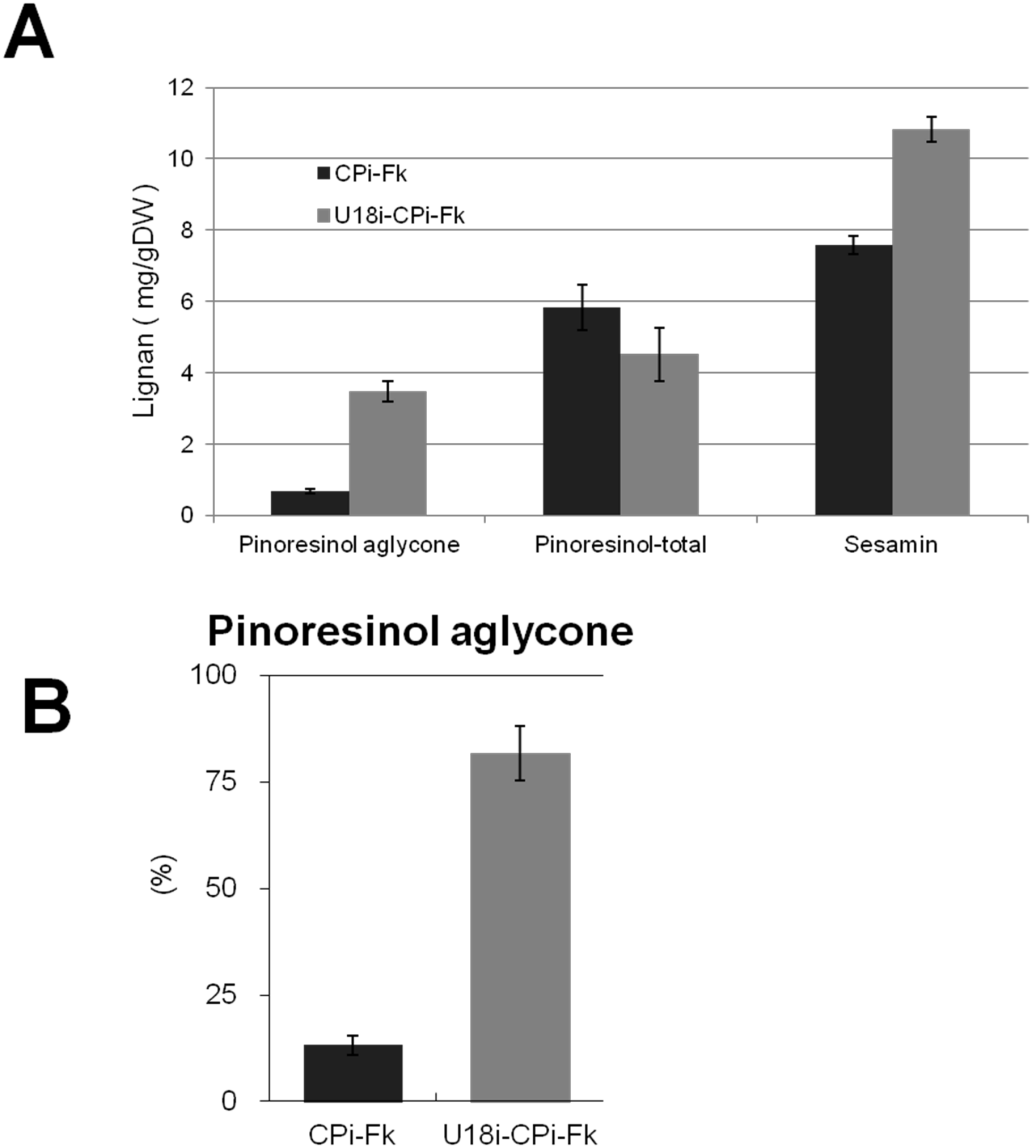
Amounts of pinoresinol aglycone, total pinoresinol (aglycone and glucosides), and sesamin. Comparison of the three lignans between CPi-Fk and U18-CPi-Fk (A). The ratio of pinoresinol aglycone to total aglycone was also calculated (B). Pinoresinol aglycone was quantified separately after a culture period of 15 d under the same conditions. Values (%) in (B) are presented as the ratio of pinoresinol aglycone (2.21 ± 0.72 μg/ g DW and 0.34 ± 0.09 μg/ g DW) to total pinoresinol (3.71 ± 0.40 μg/ g DW and 2.88 ± 0.47 μg/ g DW) in CPi-Fk and U18i-CPi-Fk, respectively. Data are obtained from three independent experiments as the mean ± SEM (*P* < 0.05).

UGT71A18 is responsible for specific glucosylation of pinoresinol [25], and CYP81Q1 converts pinoresinol aglycone, but not its glucosides, into sesamin [7]. Hence, U18i-CPi-Fk, in which *UGT71A18* expression is not detected (Fig 2), is expected to accumulate more pinoresinol aglycone and sesamin than CPi-Fk. To evaluate the effect of UGT71A18-RNAi on the production of pinoresinol aglycone and sesamin, we assessed major lignans in CPi-Fk and U18i-CPi-Fk. The lignan mixtures extracted from 2-week cultures of each cell line were either treated or left untreated with β-glycosidase and then subjected to HPLC analysis. As shown in Fig 3A, the amount of total pinoresinol (pinoresinol aglycone and glucoside) was slightly decreased in U18i-CPi-Fk than in CPi-Fk, whereas U18i-CPi-Fk accumulated approximately 5-fold higher amounts of pinoresinol aglycone (2.21 ± 0.72 μg/ g of cell dry weight, DW) than CPi-Fk (0.34 ± 0.09 μg/ g DW). Furthermore, as depicted in Fig 3B, the ratio of pinoresinol aglycone to total pinoresinol in U18i-CPi-Fk is 81.81 ± 6.43%, which is approximately 6.5-fold greater than that in CPi-Fk (13.19 ± 2.35%). These results prove that the introduction of UGT71A18-RNAi construct contributed a great deal to the increase in the ratio of pinoresinol aglycone to total pinoresinol. Consistently, the lignan quantification (Fig 3A) revealed that the amount of sesamin in U18i-CPi-Fk (10.83 ± 0.35 μg/ g DW) was also approximately 1.4-fold greater than that in CPi-Fk (7.57 ± 0.27 μg/ g DW). Collectively, these results verified that U18i-CPi-Fk had considerably higher pinoresinol aglycone and sesamin productivity than the prototypic cell, CPi-Fk, due to the introduction of UGT71A18-RNAi.

### Effects of light on lignan production in U18i-CPi-Fk

**Fig 4:**
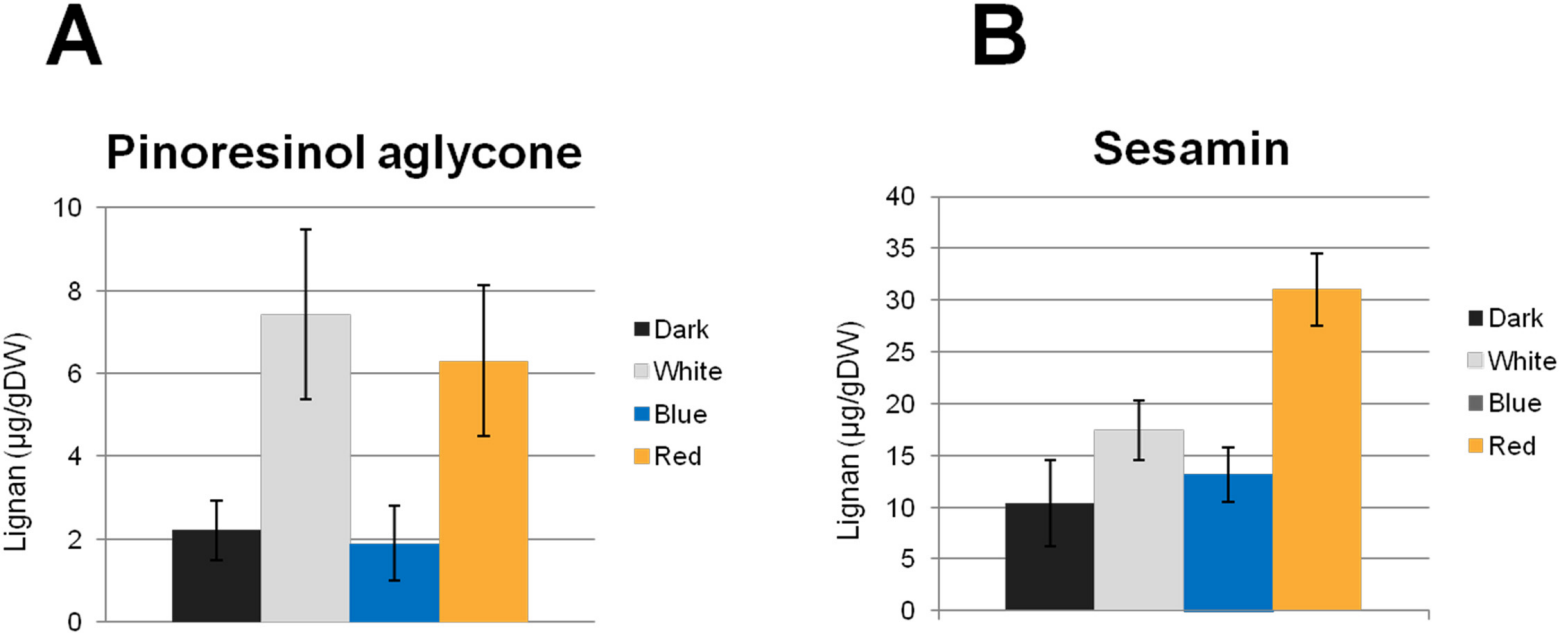
Comparison of the production of (A) pinoresinol and (B) sesamin in U18i-CPi-Fk cells under the dark, white, blue, and red continuous light conditions. Lignans were quantified separately for each sample after a culture period of two weeks in the same medium. Data are presented as the average of three independent experiments (mean ± S.E.M., *P* < 0.05).

**Fig 5:**
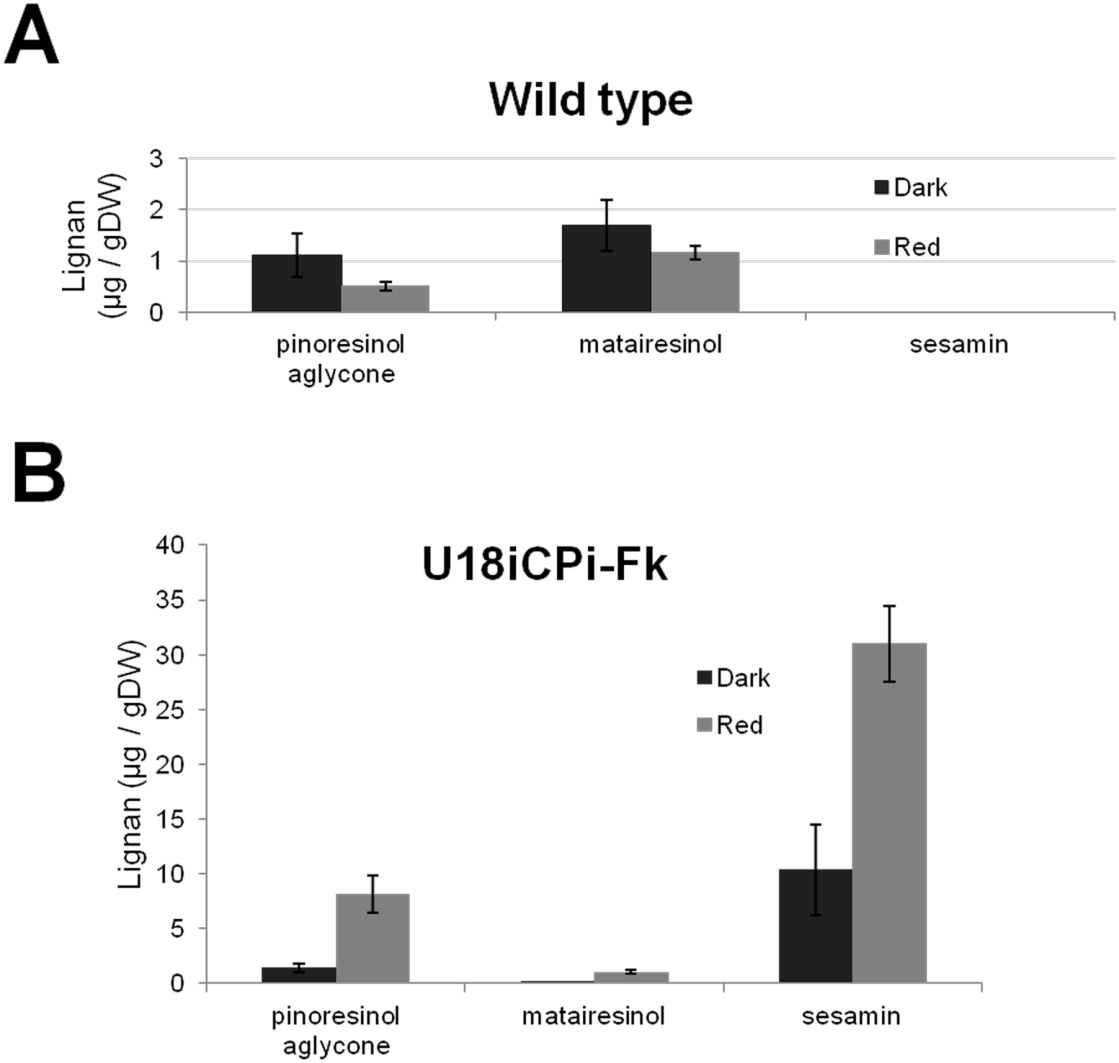
Differential effects of red LED irradiation on the increase of lignan production in *Forsythia* wildtype and U18i-CP-Fk. Each indicated lignan in wildtype (**A**) and U18i-CP-Fk (**B**) in the dark (black) and red LED (gray) was assessed. Each point represents the mean ± S.E.M (*P* < 0.05) of three preparations.

Productivity of several plant specialized metabolites including lignans is affected by the wavelength and the period of light [29–32]. To examine whether the light quality alters lignan production in U18i-CPi-Fk, we compared lignan content in U18i-CPi-Fk cultured either under the dark condition, white fluorescent, blue LED, or red LED light for two weeks following two-week pre-culture under the dark condition. As shown in Fig 4A, pinoresinol aglycone was 3.4-fold (7.41 ± 0.06 μg/ g DW) and 2.8-fold (6.30 ± 1.82 μg/ g DW) greater produced under white fluorescent and red LED, respectively, than under the dark condition (2.21 ± 0.72 μg/ g DW). A striking feature is that sesamin production in U18i-CPi-Fk was approximately 3-fold (31.02 ± 3.45 μg/ g DW) up-regulated specifically under red LED, whereas neither white fluorescent nor blue light resulted in elevation of sesamin production (Fig 4B). To examine the specificity of the effect of red LED on the lignan production in U18i-CPi-Fk, we compared accumulation of pinoresinol aglycone and matairesinol aglycone in the *F*. *koreana* wildtype cells and U18i-CPi-Fk. In wild-type cells cultivated under red LED, the levels of pinoresinol aglycone and matairesinol aglycone were decreased to 50% (0.51 ± 0.09 μg/ g DW) and 80% (1.16 ± 0.14 μg/ g DW), respectively, compared to the amounts of pinoresinol (1.02 ± 0.18 μg/ g DW) and matairesinol (1.45 ± 0.20 μg/ g DW) in cells cultured in the dark (Fig 5A). In contrast, red LED light increased the production of these lignans and sesamin in U18i-CPi-Fk relative to production in cells cultivated in the dark; production of pinoresinol aglycone and matairesinol aglycone were approximately 3-fold (8.13 ± 1.68 μg/ g DW) and 2.5-fold (1.07 ± 0.14 μg/ g DW), respectively, under red LED irradiation than those in the dark (Fig 5B). Taken together, these data provide compelling evidence for the specific effect of red LED on the increase of sesamin production in U18i-CPi-Fk.

### Cryopreservation and recovery of U18i-CPi-Fk

**Fig 6:**
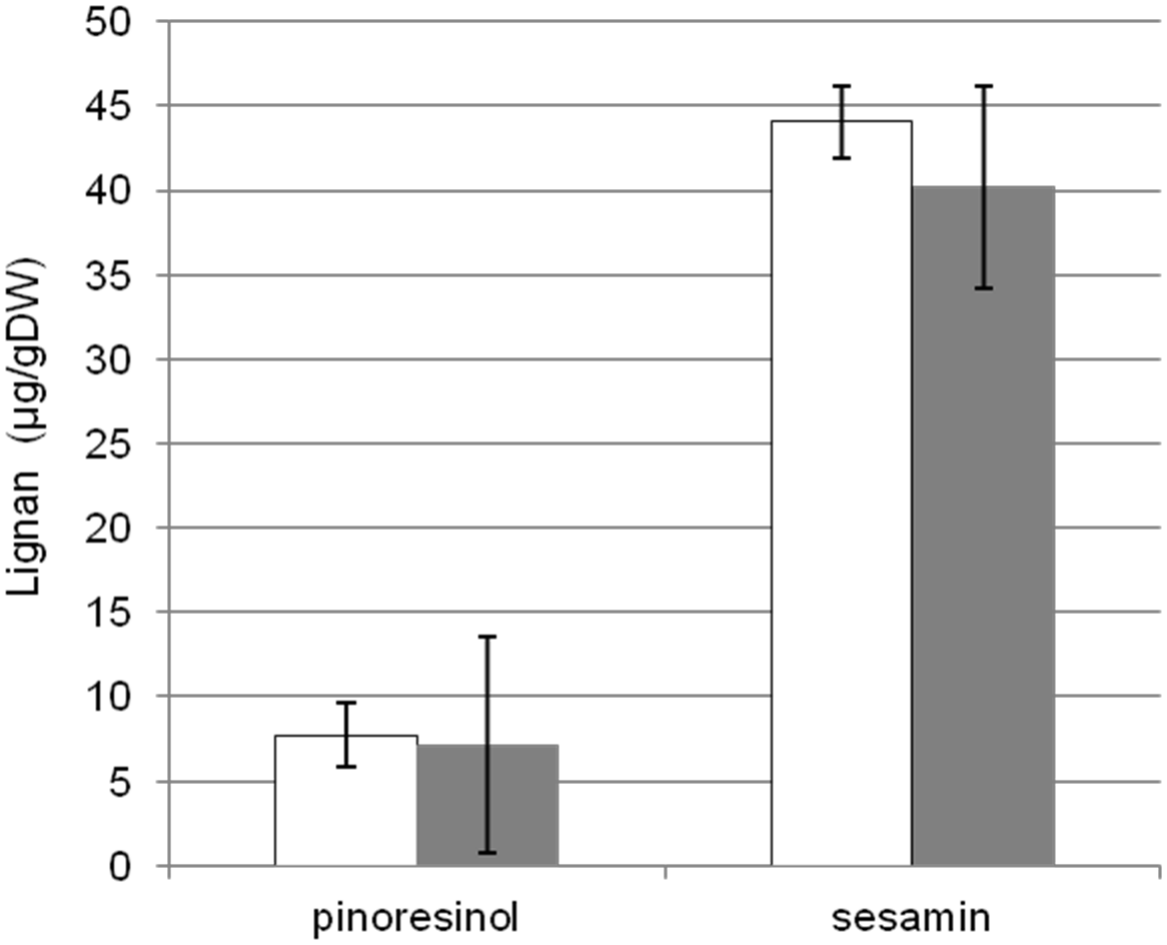
Evaluation of lignan production after cryopreservation of U18i-CPi-Fk. Pinoresinol aglycone and sesamin were extracted from U18-CPi-Fk before cryopreservation (white) and after re-thawing following 6-month cryopreservation (gray). Each lignan was assessed separately after 55-day culture since the recovery under the same condition and expressed as the mean ± SEM of three independent experiments (*P* < 0.05).

Continuous culturing of transgenic plant cells frequently resulted in the loss of transgene functions or reduced cell viability. Recently, cryopreservation procedures for various plant cells have been developed [27, 28]. We thus attempted to establish a procedure for sodium alginate-based cryopreservation of *F*. *koreana* cell cultures under various conditions. Although we failed to preserve or revive any *Forsythia* cells using reported procedures, U18i-CPi-Fk was cryopreserved without significant loss of viability for more than six months as described in the Materials and Methods section. Notably, productivity of both pinoresinol aglycone and sesamin of U18i-CPi-Fk re-cultured for 55 days following six-month cryopreservation was comparable to those before cryopreservation (Fig 6). These data lead to the conclusion that U18i-CPi-Fk is an excellent long-term sustainable platform for lignan production.

## Discussion

Metabolic engineering using transgenic organisms is one of the most promising strategies for efficient and sustainable production of beneficial, but rare, natural compounds in nature. Transgenic plant cells have prominent advantages over transgenic microbes or animals for producing such phytochemicals, including sesamin. Most of the biosynthetic pathways for plant specialized metabolites involve multiple enzymatic steps that are unique to selected plant species. Therefore, transformation of a whole set of the biosynthetic genes would be required for the establishment of transgenic microbial or animal cell lines that could produce phytochemicals [33–36]. In contrast, transgenic plants or their cell lines producing phytochemicals of interest can be generated by transformation of a minimum set of genes using a plant species that originally biosynthesizes precursors or metabolites structurally related to the target compound. Nevertheless, introduction of more than two sets of genes into a plant has been markedly limited, and no such multiple-transgenic plants have yet been created for metabolic engineering of lignan biosynthetic pathways. In the present study, we show stable, efficient and sustainable production of sesamin using novel triple-transgenic *Forsythia* cells, U18i-CPi-Fk. The triple-transgenic *Forsythia* cells, U18i-CPi-Fk, produced 1.4-fold greater sesamin and 5-fold greater pinoresinol aglycone than CPi-Fk (Fig 3). To the best of our knowledge, this is the first report on the production of both an endogenous and exogenous plant specialized metabolites by triple-transgenic plants.

**Fig 7:**
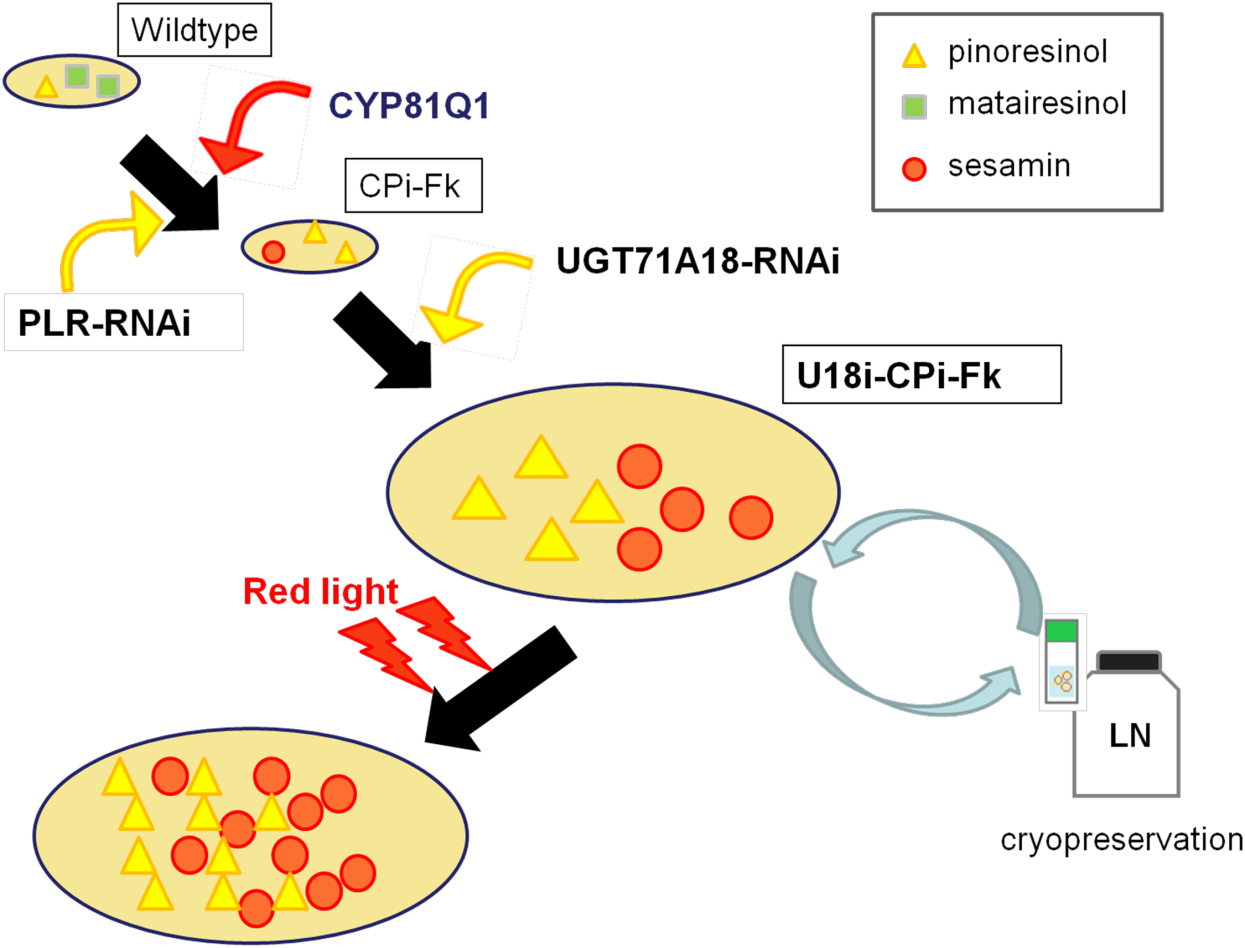
Strategy for efficient, stable, and sustainable lignan production via metabolic engineering of *Forsythia* cells.

The level of phytochemical production by plants is also regulated by various environmental factors, particularly light wavelengths [29–32]. The responsiveness of plant cell cultures and organs to light stimuli enable the regulation of the production of specialized metabolites, which cannot be used for microbes or animals. Indeed, red LED irradiation specifically increased the production of pinoresinol and sesamin up to 3-fold in U18i-CPi-Fk over production in the dark (Figs 4 and 5). The increase of sesamin is compatible with that of pinoresinol aglycone (Figs 4 and 5). In contrast, red LED irradiation resulted in the reduction of pinoresinol production in *F*. *koreana* wildtype cells (Fig 5A). These results suggest that red LED light affect the expression or functions of genes responsible for pinoresinol biosynthesis in *F*. *koreana* cells. In addition, in *Linum album* cell culture, red light reportedly up-regulates expression of enzymes involved in the biosynthesis of phytochemicals upstream of pinoresinol, including phenylalanine ammonia-lyase (PAL) and cinnamoyl-CoA reductase (CCR) [30]. Similarly, production of non-lignan plant specialized metabolites is affected by other light types [30, 37–40]. In this study, the treatment of U18i-CPi-Fk cells with white fluorescent tubes failed to promote sesamin production, while the white light significantly increased pinoresinol aglycone production (Fig 4). These results can be interpreted in two ways. First, the white light tubes include other light wavelengths that might induce metabolic processes or degradation of sesamin. Alternatively, other light wavelengths than red LED might hinder the enzyme activity of CYP81Q1. Although the precise molecular mechanisms await further investigation, these findings clearly demonstrate the importance of the choice of specific light source for maximal production of metabolites of interest in plant cell culture system. Taken together, the present data prove that red LED-directed increase in lignan productivity is a unique and promising advantage of U18i-CPi-Fk for lignan production (Fig 7).

Glycosylation affects the chemical reactivity and water solubility of plant specialized metabolites, frequently leading to changes in their biological activities and the relevant plant phenotypes. For example, changes in the colors of flowers, leaves, and fruits greatly depend on the position and structure of sugars that are attached to the aglycones of various phytochemicals [41–50]. In *F*. *koreana*, 90% of total pinoresinol is stored as glucosylated forms in vacuole [2, 20, 24], and pinoresinol glucosides are not converted into sesamin by CYP81Q1 [8]. These findings are in good agreement with the present data demonstrating that RNAi against UGT71A18 led to the marked increase in productivity of sesamin as well as pinoresinol aglycone in U18i-CPi-Fk (Fig 3). In addition, we failed to generate UGT71A18-RNAi-single or UGT71A18-RNAi- and PLR-RNAi-double transgenic *Forsythia* cells, whereas generation of PLR-RNAi-*Forsythia* cells was succeeded in our previous study [20]. These results suggest that the accumulation of unreacted pinoresinol aglycone exhibit cytotoxicity that is more lethal to *Forsythia* than the loss of other lignans downstream of pinoresinol that occurred due to the introduction of PLR-RNAi. In other words, the toxicity of pinoresinol aglycone reinforces the biological significance of pinoresinol glucosylation in *Forsythia*. Such a view is further supported by previous reports of the toxicity of pinoresinol and its metabolites on plants [51–53]. In contrast, the introduction of CYP81Q1 resulted in increased conversion of pinoresinol aglycone to sesamin and likely contributed to a reduction in the toxicity associated with pinoresinol aglycone accumulation, eventually leading to the generation of U18i-CPi-Fk.

Resilient platforms for the production of beneficial materials in cell culture systems require not only high and efficient productivity but also protocols for long-term and reproducible stocks without significant loss of cell viability. In the present study, we show that, after cryopreservation for six months in liquid nitrogen using a sodium alginate preparation method, U18i-CPi-Fk successfully propagated and produced pinoresinol and sesamin at levels comparable to the original cultures (Fig 6). Universal procedures for long-term stock of plant cell cultures, unlike those of seeds or animal cell cultures, have not been well established. Moreover, cryopreservation procedures for a particular plant species are not readily applicable to other species [27, 28, 54]. In addition, long-term cultures of plant cell cultures frequently result in the loss of cell viability or of inserted genes. In our previous study, we observed a decrease in the growth rate of CPi-Fk cells after two years of culture, and eventually, proliferation loss (data not shown). Consequently, the present establishment of the freeze stocks of U18i-CPi-Fk endorses the usefulness of U18i-CPi-Fk as a stable and sustainable platform of lignan production anytime and anywhere.

Model plants, such as *A*. *thaliana*, *Nicotiana tabacum*, and *Oryza sativa*, are frequently endowed with only partial or no biosynthetic pathway for specialized metabolites of interest, or, if any, can biosynthesize only very small amounts of most specialized metabolites including lignans [5, 7]. Hence, generation of transgenic model plants competent in lignan production would require complexed transformation of multiple exogenous genes. Indeed, no studies have ever reported efficient and sustainable production of specialized metabolites such as lignans using these model plants. Consequently, a transgenic metabolic engineering strategy using *Forsythia*, which biosynthesizes large amounts of a basal lignan, pinoresinol (Fig 1), is one of the most promising for efficient and sustainable production of lignans (Fig 7). Moreover, the pinoresinol aglycone to total pinoresinol ratio is high in U18i-CPi-Fk (Fig 3B) owing to the gene-silencing of a pinoresinol-glucosylating enzyme, UGT71A18 (Fig 2). Recent studies of genomes and transcriptomes of non-model, lignan-rich plants such as *Sesamum* [55–57], *Linum* [58–60], and *Podophyllum* [61–63] will enhance not only the elucidation of species-specific total lignan biosynthetic pathways, but also facilitate metabolic engineering of lignan production through transformation of *Forsythia* plants and cell cultures with newly identified lignan biosynthetic enzyme genes. In addition, a growing body of studies has revealed that the biosynthesis of lignans, including pinoresinol, lariciresinol, and podophyllotoxin, is potentiated by chemical and fungal elicitors in a plant/fungal species- and compound-specific fashion in *Linum* and *Podophyllum* cells [6, 7]. Particularly, various elicitors have been found to upregulate the gene expression of early lignan-biosynthetic enzyme genes: *CCR*, *PAL*, and *PLR*, which ultimately contributes to an increase in production of lariciresinol and podophyllotoxin in *Linum* [6, 7, 64–68]. Although the current sesamin content in U18i-CPi-Fk cells with red LED irradiation (Fig 4) is still low compared to that in sesame seed oil (0.4–0.6% w/w) [1–7], the productivity of sesamin in U18i-CPi-Fk will be more efficient than that of sesame seed oil by combination of the aforementioned multiple approaches for the up-regulation of lignan biosynthesis and the advantages of scalability and year-round sesamin extraction periods of U18i-CPi-Fk. Collectively, we conclude that U18i-CPi-Fk possesses unprecedented functionalities for transgenic metabolic engineering-based platforms for the efficient production of an exogenous lignan, sesamin, and has potential for producing other beneficial lignans via metabolic engineering of the relevant biosynthetic pathways. Consequently, U18i-CPi-Fk is a novel platform that will pave the way for the conversion of conventional agricultural lignan production to innovative bio-industrial lignan production

## Abbreviations

CCR: cinnamoyl-CoA reductase
DIR: dirigent protein
LC-MS: liquid chromatography-mass spectrometry
PAL: phenylalanine ammonia-lyase
PLR: pinoresinol/lariciresinol reductase
RNAi: RNA interference
RT-PCR: reverse transcription-PCR
SDG: secoisolariciresinol diglucoside
